# 3 = 1 + 2: how the divide conquered de novo protein structure prediction and what is next?

**DOI:** 10.1093/nsr/nwad259

**Published:** 2023-10-03

**Authors:** Yaoqi Zhou, Thomas Litfin, Jian Zhan

**Affiliations:** Institute of Systems and Physical Biology, Shenzhen Bay Laboratory, China; Institute for Glycomics, Griffith University, Australia; Institute for Glycomics, Griffith University, Australia; Institute of Systems and Physical Biology, Shenzhen Bay Laboratory, China

Protein structure prediction has been a challenging problem for >60 years. Its progress has been considered incremental for the past 20 years until leaps were made by AlphaFold that seemed to suddenly reach experimental accuracy. We argue that this unexpected success in 3D structure prediction was foreshadowed by prior advances in predicting 1D backbone structures and 2D side-chain distance maps in continuous angle and distance space as well as the embedding of these 1D and 2D features for end-to-end learning. This divide of the 3D problem into 1D and 2D subproblems (3 = 1 + 2) conquered the problem of the mapping of multiple homologous sequences to their associated ‘single’ structure by using AlphaFold 2, but not the holy grail of mapping a *single* sequence to its real-world structure and dynamics. The perspective in this post-AlphaFold 2 era is discussed.

Since they were first discovered in 1838 by the Dutch chemist Gerardus Johannes Mulder, proteins have been the focus of molecular biology because of their versatile roles in every biological process. By contrast, the chemical structures of proteins are deceptively simple: linear chains made of different arrangements of 20 amino acid residues. The functional versatility of proteins was attributed to their ability to fold into unique and diverse 3D structural forms. However, experimental determination of protein structures is tedious, costly and labor-intensive. The discovery that protein structures are wholly determined by their sequences led to the well-known Anfinsen's thermodynamic hypothesis [1] and 60 years of efforts to computationally fold and predict protein structures given their sequences.

The progress of these efforts has been recorded according to Global Distance Test (GDT) scores since 1994 by biennial meetings of the critical assessment of structure prediction techniques (CASP) [2]. Twenty years of effort only led to a small increase in the trend lines represented by the overall GDT score from ∼27 to ∼32 for the most difficult targets (without any known templates) from 1996 to 2016 [2]. At this pace, it would take another 212 years to reach experimental accuracy (GDT > 85). Unexpectedly, AlphaFold [3], developed by DeepMind, made the first leap to ∼65 at CASP 13 in December 2018, followed by the second leap to ∼85 by AlphaFold 2 [4] that removed the remaining gap from experimentally determined structures for most structures predicted at CASP 14 in December 2020. As the revolution caused by AlphaFold reverberates in every corner of biology, it is time to reflect on how ‘sudden’ leaps occurred in a not-so-sudden manner. This retrospective analysis can provide us with a perspective on where this revolution will take us next.

## DIVIDING PROTEIN STRUCTURE PREDICTION

**Figure 1.**
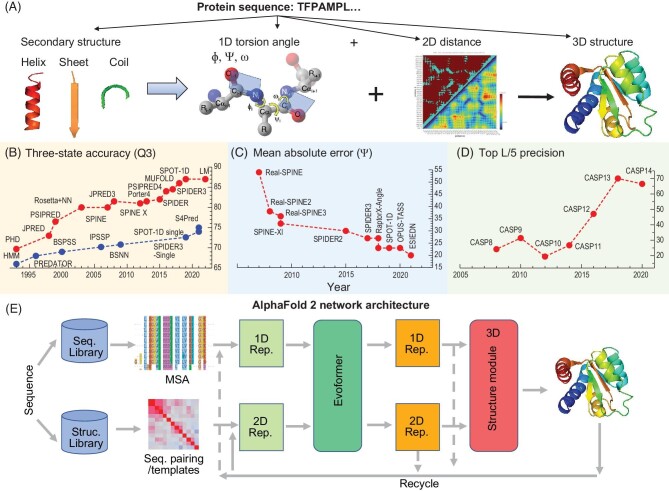
(A) The division of the protein structure prediction problem into the subproblems of predicting 1D backbone structures (in discrete secondary structures or continuous torsion angles) and 2D distance maps for use in 3D structure prediction (3 = 1 + 2). (B) Three decades of progress since PHD (profiling network from HeiDelberg) first employed evolution information for three-state secondary structure prediction in 1993 that approached the theoretical limit in around 2018 by using SPIDER3, MUFOLD and SPOT-1D (shown in red). By comparison, for single-sequence-based methods (without evolution information, shown in blue), the progress is much slower at ∼74% with the most recent methods (SPOT-1D-Single and S4PRED). (C) Reduction in mean absolute errors of real-value prediction of *ψ* angles from 54° to 20° in 15 years of effort in predicting real-value torsion angles. (D) The performance of the top predictors in terms of the precision for top L/5 predictions for free modeling targets from CASP 8 to CASP 14 indicating the leap in accuracy in 2016 and 2018. (E) AlphaFold 2 implemented the ‘3 = 1 + 2’ approach in an end-to-end fashion without an explicit energy function.

Direct prediction of the 3D structure from a protein sequence faces the challenge of huge conformational space and lack of an accurate free energy function. Thus, this problem has long been divided into several simpler subproblems, as shown in Fig. 1A, including prediction of the 1D backbone structure (in secondary structure or torsion angles) and 2D distance maps, with the hope of assisting in 3D structure prediction. This divide-and-conquer approach can be viewed as a ‘3 = 1 + 2’ approach.

## THE ‘1’: BACKBONE STRUCTURE IN DISCRETE SECONDARY STRUCTURE AND CONTINUOUS TORSION ANGLES

Backbone structure prediction has been dominated by the prediction of three-state secondary structures. As shown in Fig. 1B, the methods based on a single protein sequence are not that accurate, at ∼74% three-state accuracy (Q_3_), even with the most advanced deep-learning techniques such as SPOT-1D-Single and S4PRED. One major advance in the three-state prediction was the use of evolutionary profiles by profile network from HeiDelberg (PHD) in 1993, which allowed gradual improvement to 87% by using our method, SPOT-1D, in 2019, within the theoretical limit [5]. As shown in Fig. 1B, there was a fast pace of accuracy improvement in around 2016 due to the use of increasingly improved deep-learning algorithms in SPIDER, PSIPRED and MUFOLD. (Due to space limitations, all citations to various named methods are provided in the Supplementary material.)

However, discrete secondary structure states have the following drawbacks when employed as restraints in 3D structure prediction: ideal helices and sheets do not exist, and predicted coil states do not contain structural information. By comparison, backbone structures can be constructed directly if continuous real values of three backbone torsion angles (*φ, ψ* and *ω*) are known. Given that *ω* is often fixed at 180°, we made the first attempt to predict real values of both torsion angles (*φ* and *ψ*). Initial errors represented by *ψ* angles by using Real-SPINE were huge (the mean absolute error was 54°). As shown in Fig. 1C, this situation was improved to 23° by taking care of angle periodicity (Real-SPINE2), improved neural networks, mixed prediction of discrete and continuous real values (SPINE-XI) [6], followed by a number of methods based on deep learning with the reduction of error by more recent work (ESIDEN) to 20°. More importantly, it was demonstrated, as early as 2009, that these real-value predicted angles were accurate enough to be useful to generate non-ideal helical and strand structures as well as coiled structures for improving 3D structure prediction [6]. In retrospect, the move from discrete to continuous prediction in backbone structures enabled fragment-free protein structure prediction with a protein-specific energy function [6,7] as well as end-to-end structure prediction through differentiable learning, as seen below.

## THE ‘2’: SIDE-CHAIN DISTANCE MAP

Another subproblem is to predict 2D discrete contact or continuous-distance maps of Cα atoms or side-chain Cβ atoms. Unlike secondary structures, contact map prediction has been a part of the CASP assessment since CASP 2 (1996). Its assessments were typically conducted for new folds or free modeling targets. Figure 1D shows the progress of top performers in terms of the precision of top L/5 predictions from CASP 8 to CASP 14 for long-range contacts. As one can see, similarly to backbone structure prediction, there is a great improvement in contact prediction in around 2016 (CASP12), as a result of combining deep learning with direct coupling analysis such as in PconsC2 and RaptorX-Contact. Prediction of discrete two-state contact maps has further evolved into distance-grid maps or even continuous-distance prediction as the power of deep learning continues to increase [8]. During the transition from discrete to continuous prediction in backbone structures, discrete-to-continuous-distance prediction paved the way for placing the whole ‘3 = 1 + 2’ approach in the continuous and differentiable space.

## THE ‘3’: EVOLUTION OF DE NOVO STRUCTURE PREDICTION PRIOR TO ALPHAFOLD

Protein structure prediction was started from homology or template-based modeling (Supplementary Table S1). Due to the lack of recognizable templates for most proteins, the methods for template-free modeling (free modeling in CASP, also called de novo structure prediction) were actively developed. Mini-template or fragment-based techniques were first proposed by Bowie and Eisenberg [9] and dominated over the de novo protein structure prediction for the next 20 years with representative methods such as Rosetta, I-TASSER, QUARK, SimFold and FragFold. Fragment-based sampling techniques map sequence fragments onto structural fragments in the fragment libraries for energy-guided structural assembly. Employing rigid fragments allowed faster samples of protein-like conformations, but locating the best fragments is challenging and the perfect matches between the native fragments and library fragments may not exist.

A more natural way to fold a protein is template/fragment-free modeling. This approach started with physical-based approaches (*ab initio* structure prediction) but with limited successes for peptides and small proteins only [7]. Knowledge-based fragment-free methods were developed to employ torsion-angle sampling bias [10] such as in FB5-HMM and CRFSampler or predicted torsion angles as protein-specific scoring/restraints such as in our effort (SPINE-XI [6]). However, these nascent fragment-free approaches, in particular, whereby predicted torsion angles were used as protein-specific scoring/restraints [7], were marginalized due to the success of fully developed fragment-based approaches in CASP meetings, despite the fact that predicted torsion angles are increasingly more accurate and useful to generate backbone structures including coil regions since SPINE-XI in 2009 [6].

## ALPHAFOLD: A FRAGMENT-FREE, PROTEIN-SPECIFIC SCORING METHOD

AlphaFold [3] broke away from the dominant fragment-based methods by developing a fragment-free, protein-specific scoring method, which achieved #1 in CASP 13 (2018). Similar approaches are CONFOLD and COINFOLD but with discrete secondary structure and contact maps. Our method SPOT-Fold employed predicted continuous angles but with discrete predicted contact maps. Grid-based prediction of torsion angles were also employed in SPIDER2-Grid. Xu also employed both predicted angles and predicted distances as protein-specific energy terms [8]. AlphaFold, on the other hand, predicted both residue–residue distance and backbone torsion-angle distributions and converted both as a protein-specific scoring functions for minimization. AlphaFold showed that ‘the gradient-descent (fragment-free) method that was used later in CASP performed better than the fragment assembly method, in each category’ [3], confirming the advantage of the fragment-free approach with a protein-specific energy function [7]. The winning of CASP by AlphaFold by a large margin over other methods in CASP 13 was a significant event as it finally brought the attention of the scientific community to the fragment-free technique with a protein-specific score, initiated nearly 10 years ago [6]. This progress was made by following the 3 = 1 + 2 approach in a nearly continuous grid space.

## ALPHAFOLD 2: END-TO-END LEARNING BY EMBEDDING 3 = 1 + 2

AlphaFold 2 [4] in CASP 14 continues AlphaFold's fragment-free approach. It, however, no longer employed an energy function directly, as shown in Fig. 1E. (Although it utilized an AMBER force field for relaxing atomic clashes in the last step, it did not change the structures predicted from the end-to-end model in any significant way.) Sequence-to-structure mapping was all done within the neural network. With this end-to-end setting, AlphaFold 2 employed homologous sequences directly as an input to extract 1D and 2D evolutionary features and directly predicted global affine transformations for rigid backbone reference frames and local side-chain torsion angles to facilitate all-atom structure assembly. Moreover, predicted structures were updated in the structure module as well as in model recycling. Thus, AlphaFold 2 bears no resemblance to AlphaFold in choosing the ‘1’ and the ‘2’ to produce the ‘3’.

The network architecture of AlphaFold 2 did not come from nowhere. The end-to-end prediction was enabled by the emergence of back-propagation for any differentiable loss function in computer science 2 years early (2016) [11]. The first end-to-end protein structure predictor, RGN (recurrent geometric network), employed one-shot structure prediction by constructing structures from predicted torsion angles (1→3), which is the same as the fragment-free approach in SPINE-XI except that SPINE-XI employed the energy optimization outside the neural network [6,7]. At nearly the same time, NEMO (neural energy modeling and optimization) was developed by iteratively refining an initial structure guess through considering both the backbone angles and the pairwise distances of intermediate structures (1 + 2→3). Both RGN and NEMO explicitly enforced the chain constraint by sequentially constructing tertiary coordinates using predicted backbone torsion angles. AlphaFold 2 resembles NEMO in many ways, including the use of local reference frame representations for each amino acid residue, 1D and 2D intermediate representations, consideration of rotational and translational invariance, and integration of structure optimization in model learning. However, unlike RGN and NEMO, the unique features of AlphaFold 2 that are most critical for its outstanding performance are as follows. First, AlphaFold 2 generated tertiary structures by predicting independent, residue-level, affine transformations for rigid backbone frames—likely avoiding the propagation of incremental errors and the traps of local minima. Second, AlphaFold 2 has turned the problem of mapping a single sequence to a single structure to the problem of mapping multiple homologous sequences to a single structure. In this way, multiple homologous sequences were input together so that co-evolution information can be captured more directly, unlike the pre-processed 1D sequence profiles employed in RGN and NEMO that likely lost most of the co-evolution information. Furthermore, AlphaFold 2 employed the huge sequence data set (Big Fantastic Database, BFD) and most structures in the protein databank (PDB) (170 000) to train a large model. The computational resource for training this huge model was beyond the reach of most academic groups.

Since the success of AlphaFold 2, a number of end-to-end methods were developed, including RoseTTAFold, RGN2, ESMFold and OmegaFold, in addition to OpenFold and UniFold, which are direct reimplementations of the AlphaFold2 model. Most new methods focus on the use of language models (LMs) for structure prediction. Implicit modeling of evolutionary information can dramatically accelerate inference speeds but has typically been accompanied by a drop in modeling accuracy—particularly for small protein families. Table 1 provides a summary of these new methods and highlights the key aspects for each method. Only RoseTTAFold2 and OmegaFold report comparable performance to AlphaFold2. These methods, however, did not seem to gain much over AlphaFold 2 based on the results released from CASP 15 (see CASP 15 abstract at https://predictioncenter.org/casp15/doc/CASP15_Abstracts.pdf) as the best-performing methods relied on AlphaFold 2 as a part of their results.

**Table 1.** Description of end-to-end protein structure predicting models developed in the wake of AlphaFold2.

Method	Year	LM^a^	Description
RoseTTAFold (RF1)	2021	N	Biaxial attention for MSA^a^ processing—more computer-efficient than triangle attention. SE(3)-equivariant transformer for structure module
RGN2	2022	Y	LM^a^ representations directly input to structure module. Structures iteratively constructed from predicted internal coordinates—explicitly enforces chain continuity. Offline structure refinement
OmegaFold	2022	Y	Geoformer module for processing LM^a^ representations—strictly maintains geometric consistency compared with triangle attention. IPA^a^ for structure module. Curriculum learning to prioritize challenging structures
RoseTTAFold2 (RF2)	2023	N	Biaxial attention for MSA^a^ processing biased by structures from parallel 3D track. Adopted many AF2^a^ strategies including FAPE^a^ loss, recycling and distillation. Natively supports complexes in a unified model
ESMFold	2023	Y	Evoformer module for processing LM^a^ representations. IPA^a^ for structure module. Highly optimized LM^a^ hyperparameters

^a^LM, language model; MSA: multiple sequence alignment; IPA, invariant point attention; AF2, AlphaFold2; FAPE, frame-aligned point error.

Supplementary Table S1 provides a list of historically representative methods that show the evolution of protein structure modeling from template-based, fragment-based to fragment-free in one dimension and alignment-based, energy-based, to end-to-end prediction in the other dimension.

## WHAT IS NEXT?

Dividing of the protein structure prediction into 1D and 2D properties seems to have conquered the 3D structure prediction problem, or has it? As demonstrated above, AlphaFold 2 has only solved the problem of mapping multiple homologous sequences to one single structure. As a result, the prediction of AlphaFold 2 is accurate only for those proteins with an abundance of homologous sequences. Using protein LMs such as in RGN2 or ESMFold is not the solution because LMs implicitly learn from multiple closely related sequences. Employing ‘natural’ homologous sequences also means that AlphaFold 2 is unable to predict deleterious structural variations. In fact, it is known that even a single mutation could lead to large structural changes and many human diseases resulted from single missense mutations [12]. Thus, the ultimate challenge is to determine the correspondence between one sequence and its structure. We are far from the resolution of this problem because the ∼74% accuracy for single-sequence-based prediction of secondary structures is far from the theoretical limit of 86%–90% (Fig. 1B). Thus, to advance our ability to predict the protein structure from a single sequence, we first must demonstrate that we can predict secondary structures with ∼86% accuracy at the single-sequence level. Given a lack of large data for structural differences between homologous sequences, physics-guided learning techniques [13] could be an effective way to reduce the data required for single-sequence-based prediction.

If a single-sequence-based prediction for a static structure is yet to be available, it will be even more challenging to predict protein dynamics, conformational transition, mutation-induced changes in protein stability and protein–ligand interactions by using deep learning. This is because the predictions of the latter properties are even more single-sequence-specific and close homologous sequences may well act differently in terms of dynamics, stability and interactions with small ligands. Incremental improvements rather than significant advances are expected in the near future.

On the other hand, protein–protein complex structure prediction and protein design are the research areas that have seen and will continue to see more significant advances soon. Protein design, an inverse problem of protein structure prediction, was dominated by the energy-based methods until the development of SPIN in 2014—the first neural-network-based technique for predicting the probability of amino acid types at each sequence position directly from structures. SPIN enabled protein design by selecting the sequence of the highest probable amino acid type at each position [14]. AI-driven protein design now has become the mainstream and has progressed from fixed backbone design, flexible design based on sequence-based structure prediction (hallucination) to structure and sequence generators. Meanwhile, prediction of protein–protein complex structures may well be addressed soon by better harnessing co-evolution information from multiple homologous sequences if available [15].

Another relevant research area is RNA structure prediction from RNA sequences. The techniques for RNA structure prediction were recently assessed in CASP 15. Despite 18/27 of the participating methods being AI-based, the top 4 performing techniques all used the traditional energy-based approach. The top predictor was based on a newly developed statistical energy function, BRiQ, tailored to capture orientation dependence of nucleobase-centric interactions. The failure of AI-driven RNA structure prediction in CASP15 highlights the challenges that are unique for the application of deep learning to RNA structure prediction. These challenges are due to the limited information from four-letter coded sequences, the lack of sequence conservation, the complexity of backbone torsion angles and the scarcity of RNA structures in protein databanks (only 3%). Nevertheless, some advances in RNA full base-pairing structures were made with methods such as SPOT-RNA, SPOT-RNA2, RNAcontact and SPOT-RNA-2D. With more data and better algorithms, AI-driven RNA structure prediction will be surely back in the spotlight in the near future.

In summary, the success of AlphaFold 2 in mapping homologous sequences to a single structure is not the end of structural biology, but the beginning of an era that can address many more exciting problems than previously thought possible.

Note: Due to space limitations, all methods mentioned above but not cited are cited in the Supplementary material. We also apologize for any unintentional omissions due to the space limitations.

## Supplementary Material

nwad259_Supplemental_File
