# Supplementary material for: 3 = 1 + 2: how the divide conquered de novo protein structure prediction and what is next?
Source: Natl Sci Rev. 2023 Oct 3;10(12):nwad259. doi: 10.1093/nsr/nwad259 (PMC10684263; doi:10.1093/nsr/nwad259)
Supplement: nwad259_Supplemental_File [file nwad259_supplemental_file.docx]

**Supplementary Material for “3=1+2: How the Divide Conquered de Novo Protein Structure Prediction and What’s Next?”**

Yaoqi Zhou^1,2^, Thomas Litfin^2^, and Jian Zhan^1^

^1^Institute of Systems and Physical Biology, Shenzhen Bay Laboratory, Shenzhen, Guangdong Province, China

^2^Institute for Glycomics, Griffith University, Southport, Queensland 4222, Australia

**Supplementary Table 1**. Evolution of protein structure modelling from template-based, fragment-based to fragment-free in one dimension and alignment-based, energy-based, to end-to-end prediction at the other dimension. This table was not intended to be an exhaustive list. Only representative early techniques were listed here from historical perspectives.

|  | **Template-based** | **Fragment-based** | **Fragment-free** | |
| --- | --- | --- | --- | --- |
|  |  |  | **Sampling bias** | **Protein-specific scoring** |
| **Alignment-based:**  **Homology modelling** | MODELLER (1995), SWISS-MODEL (1996), BLAST/PSI-BLAST (1997), SAM-T98 (1998), HMMER (1998), FFAS (2000), and HHpred (2005). |  |  |  |
| **Alignment-based:**  **Threading/Fold recognition** | PROSPECT (2000), PROSPECTOR (2001), Raptor (2003)), SAM-T02 (2003), SPARKS (2004), TASSER (2004), SPARKS-X (2011), Raptor-X(2011), CNFpred (2012). |  |  |  |
| **Energy score-based** |  | Rosetta (1997),  FragFold (2001), SimFold (2003), I-TASSER(2007)*,* QUARK (2012) | FB5-HMM (2006), CRFSampler (2008) | SPINE-XI (2009), CONFOLD (2015), COINFOLD (2016), SPOT-Fold (2020), AlphaFold (2020)，CopulaNet (2021) |
| **End-to-End** |  |  |  | RGN (2019), NEMO (2019), AlphaFold 2 (2021), RoseTTAFold (2021), RGN2 (2021), ESMFold (2022), OmegaFold (2022),  OpenFold (2022),  UniFold (2022),  RoseTTAFold2 (2023) |

AlphaFold: Senior, A. W. et al. Improved protein structure prediction using potentials from deep learning. Nature 2020, 577, 706–710.

AlphaFold 2: Jumper, J. et al. Highly accurate protein structure prediction with AlphaFold. Nature 2021, 596, 583–589.

BLAST: Altschul, S. F.; Madden, T. L.; Schäffer, A. A.; Zhang, J.; Zhang, Z.; Miller, W.; Lipman, D. J. Gapped BLAST and PSI-BLAST: A New Generation of Protein Database Search Programs. Nucleic Acids Res 1997, 25 (17), 3389–3402. <https://doi.org/10.1093/nar/25.17.3389>.

BRiQ: P. Xiong, P.; Wu, R.; Zhan, J.; Zhou, Y. Pairing a high-resolution statistical potential with a nucleobase-centric sampling algorithm for improving RNA model refinement, Nature Communications, 2021, 12，2777.

BSNN: Bidargaddi, N. P.; Chetty, M.; Kamruzzaman, J. Combining Segmental Semi-Markov Models with Neural Networks for Protein Secondary Structure Prediction. Neurocomputing 2009, 72 (16), 3943–3950. https://doi.org/10.1016/j.neucom.2009.04.017.

BSPSS: Schmidler, S. C.; Liu, J. S.; Brutlag, D. L. Bayesian Segmentation of Protein Secondary Structure. J Comput Biol 2000, 7 (1–2), 233–248. https://doi.org/10.1089/10665270050081496.

CNFpred: Ma, J.; Peng, J.; Wang, S.; Xu, J. A Conditional Neural Fields Model for Protein Threading. Bioinformatics 2012, 28 (12), i59-66. https://doi.org/10.1093/bioinformatics/bts213.

COINFOLD: Wang, S.; Li, W.; Zhang, R.; Liu, S.; Xu, J. CoinFold: A Web Server for Protein Contact Prediction and Contact-Assisted Protein Folding. Nucleic Acids Res 2016, 44 (Web Server issue), W361–W366. https://doi.org/10.1093/nar/gkw307.

CONFOLD: Adhikari, B.; Bhattacharya, D.; Cao, R.; Cheng, J. CONFOLD: Residue-Residue Contact-Guided Ab Initio Protein Folding. Proteins 2015, 83 (8), 1436–1449. https://doi.org/10.1002/prot.24829.

CopulaNet: Ju, F. et al. CopulaNet: Learning residue co-evolution directly from multiple sequence alignment for protein structure prediction. Nat. Commun. 2021, 12, 2535.

CRFSampler: Zhao, F.; Li, S.; Sterner, B. W.; Xu, J. Discriminative Learning for Protein Conformation Sampling. Proteins 2008, 73 (1), 228–240. https://doi.org/10.1002/prot.22057.

DCRNN: Li, Z.; Yu, Y. Protein Secondary Structure Prediction Using Cascaded Convolutional and Recurrent Neural Networks. In Proceedings of the Twenty-Fifth International Joint Conference on Artificial Intelligence; IJCAI’16; AAAI Press: New York, New York, USA, 2016; pp 2560–2567.

DeepRIN: Fang, C.; Shang, Y.; Xu, D. Prediction of Protein Backbone Torsion Angles Using Deep Residual Inception Neural Networks. IEEE/ACM Trans Comput Biol Bioinform 2018, 10.1109/TCBB.2018.2814586. https://doi.org/10.1109/TCBB.2018.2814586.

ESIDEN: Xu, Y.-C.; ShangGuan, T.-J.; Ding, X.-M.; Cheung, N. J. Accurate Prediction of Protein Torsion Angles Using Evolutionary Signatures and Recurrent Neural Network. Sci Rep 2021, 11 (1), 21033. https://doi.org/10.1038/s41598-021-00477-2.

ESMFold: Evolutionary-scale prediction of atomic level protein structure with a language model | bioRxiv. https://www.biorxiv.org/content/10.1101/2022.07.20.500902v2 (accessed 2022-12-15).

FB5-HMM: Hamelryck, T.; Kent, J. T.; Krogh, A. Sampling Realistic Protein Conformations Using Local Structural Bias. PLoS Comput Biol 2006, 2 (9), e131. https://doi.org/10.1371/journal.pcbi.0020131.

FFAS: Rychlewski, L.; Jaroszewski, L.; Li, W.; Godzik, A. Comparison of Sequence Profiles. Strategies for Structural Predictions Using Sequence Information. Protein Sci 2000, 9 (2), 232–241. https://doi.org/10.1110/ps.9.2.232.

FragFold: Jones, D. T. Predicting Novel Protein Folds by Using FRAGFOLD. Proteins 2001, Suppl 5, 127–132. <https://doi.org/10.1002/prot.1171>.

HHpred: Söding, J.; Biegert, A.; Lupas, A. N. The HHpred Interactive Server for Protein Homology Detection and Structure Prediction. Nucleic Acids Res 2005, 33 (Web Server issue), W244–W248. https://doi.org/10.1093/nar/gki408.

HMMER: Eddy, S. R. Profile Hidden Markov Models. Bioinformatics 1998, 14 (9), 755–763. https://doi.org/10.1093/bioinformatics/14.9.755.

HMM: Asai, K.; Hayamizu, S.; Handa, K. Prediction of Protein Secondary Structure by the Hidden Markov Model. Comput Appl Biosci 1993, 9 (2), 141–146. https://doi.org/10.1093/bioinformatics/9.2.141.

I-TASSER: Wu, S.; Skolnick, J.; Zhang, Y. Ab Initio Modeling of Small Proteins by Iterative TASSER Simulations. BMC Biol 2007, 5, 17. https://doi.org/10.1186/1741-7007-5-17.

IPSSP: Aydin, Z.; Altunbasak, Y.; Borodovsky, M. Protein Secondary Structure Prediction for a Single-Sequence Using Hidden Semi-Markov Models. BMC Bioinformatics 2006, 7, 178. https://doi.org/10.1186/1471-2105-7-178.

JPRED: Cuff, J. A.; Clamp, M. E.; Siddiqui, A. S.; Finlay, M.; Barton, G. J. JPred: A Consensus Secondary Structure Prediction Server. Bioinformatics 1998, 14 (10), 892–893. https://doi.org/10.1093/bioinformatics/14.10.892.

JPRED3: Cole, C.; Barber, J. D.; Barton, G. J. The Jpred 3 Secondary Structure Prediction Server. Nucleic Acids Res 2008, 36 (Web Server issue), W197-201. https://doi.org/10.1093/nar/gkn238.

MUFOLD: Fang, C.; Shang, Y.; Xu, D. MUFOLD-SS: New Deep Inception-inside-Inception Networks for Protein Secondary Structure Prediction. Proteins 2018, 86 (5), 592–598. https://doi.org/10.1002/prot.25487.

NEMO: Ingraham, J.; Riesselman, A.; Sander, C.; Marks, D. Learning Protein Structure with a Differentiable Simulator; ICLR, 2019.

MODELLER: Šali, A.; Potterton, L.; Yuan, F.; van Vlijmen, H.; Karplus, M. Evaluation of Comparative Protein Modeling by MODELLER. Proteins: Structure, Function, and Bioinformatics 1995, 23 (3), 318–326. https://doi.org/10.1002/prot.340230306.

OmegaFold: Wu, R.; Ding, F.; Wang, R.; Shen, R.; Zhang, X.; Luo, S.; Su, C.; Wu, Z.; Xie, Q.; Berger, B.; Ma, J.; Peng, J. High-Resolution de Novo Structure Prediction from Primary Sequence. bioRxiv July 22, 2022, p 2022.07.21.500999. <https://doi.org/10.1101/2022.07.21.500999>.

OpenFold: Ahdritz, G.; Bouatta, N.; Kadyan, S.; Xia, Q.; Gerecke, W.; O’Donnell, T. J.; Berenberg, D.; Fisk, I.; Zanichelli, N.; Zhang, B.; Nowaczynski, A.; Wang, B.; Stepniewska-Dziubinska, M. M.; Zhang, S.; Ojewole, A.; Guney, M. E.; Biderman, S.; Watkins, A. M.; Ra, S.; Lorenzo, P. R.; Nivon, L.; Weitzner, B.; Ban, Y. A.; Sorger, P. K.; Mostaque, E.; Zhang, Z.; Bonneau, R.; AlQuraishi, M.; OpenFold: Retraining AlphaFold2 yields new insights into its learning mechanisms and capacity for generalization; bioRxiv, November 22, 2022. doi: https://doi.org/10.1101/2022.11.20.517210.

OPUS-TASS: Xu, G.; Wang, Q.; Ma, J. OPUS-TASS: A Protein Backbone Torsion Angles and Secondary Structure Predictor Based on Ensemble Neural Networks. Bioinformatics 2020, 36 (20), 5021–5026. https://doi.org/10.1093/bioinformatics/btaa629.

PconsC2: Skwark, M. J.; Raimondi, D.; Michel, M.; Elofsson, A. Improved Contact Predictions Using the Recognition of Protein like Contact Patterns. PLoS Comput Biol 2014, 10 (11), e1003889. <https://doi.org/10.1371/journal.pcbi.1003889>.

PhD: Rost, B.; Sander, C. Prediction of Protein Secondary Structure at Better than 70% Accuracy. J Mol Biol 1993, 232 (2), 584–599. https://doi.org/10.1006/jmbi.1993.1413.

Porter4: Mirabello, C.; Pollastri, G. Porter, PaleAle 4.0: High-Accuracy Prediction of Protein Secondary Structure and Relative Solvent Accessibility. Bioinformatics 2013, 29 (16), 2056–2058. https://doi.org/10.1093/bioinformatics/btt344.

PREDATOR: Frishman, D.; Argos, P. Incorporation of Non-Local Interactions in Protein Secondary Structure Prediction from the Amino Acid Sequence. Protein Eng 1996, 9 (2), 133–142. https://doi.org/10.1093/protein/9.2.133.

PROSPECT: Xu, Y.; Xu, D. Protein Threading Using PROSPECT: Design and Evaluation. Proteins 2000, 40 (3), 343–354.

PROSPECTOR: Skolnick, J.; Kihara, D. Defrosting the Frozen Approximation: PROSPECTOR--a New Approach to Threading. Proteins 2001, 42 (3), 319–331.

PSIPRED V1: Jones, D. T. Protein Secondary Structure Prediction Based on Position-Specific Scoring Matrices. J Mol Biol 1999, 292 (2), 195–202. https://doi.org/10.1006/jmbi.1999.3091.

PSIPRED V4: Buchan, D. W. A.; Jones, D. T. The PSIPRED Protein Analysis Workbench: 20 Years On. Nucleic Acids Res 2019, 47 (W1), W402–W407. https://doi.org/10.1093/nar/gkz297.

QUARK: Xu, D.; Zhang, Y. Ab Initio Protein Structure Assembly Using Continuous Structure Fragments and Optimized Knowledge-Based Force Field. Proteins: Structure, Function, and Bioinformatics 2012, 80 (7), 1715–1735. https://doi.org/10.1002/prot.24065.

RAPTOR: Xu, J.; Li, M.; Kim, D.; Xu, Y. RAPTOR: Optimal Protein Threading by Linear Programming. J Bioinform Comput Biol 2003, 1 (1), 95–117. https://doi.org/10.1142/s0219720003000186.

RaptorX: Peng, J.; Xu, J. Raptorx: Exploiting Structure Information for Protein Alignment by Statistical Inference. Proteins: Structure, Function, and Bioinformatics 2011, 79 (S10), 161–171. https://doi.org/10.1002/prot.23175.

RaptorX-Angle: Gao, Y.; Wang, S.; Deng, M.; Xu, J. RaptorX-Angle: Real-Value Prediction of Protein Backbone Dihedral Angles through a Hybrid Method of Clustering and Deep Learning. BMC Bioinformatics 2018, 19 (Suppl 4), 100. https://doi.org/10.1186/s12859-018-2065-x.

RaptorX-ContactMap: Wang, S.; Sun, S.; Li, Z.; Zhang, R.; Xu, J. Accurate De Novo Prediction of Protein Contact Map by Ultra-Deep Learning Model. PLoS Comput Biol 2017, 13 (1), e1005324. https://doi.org/10.1371/journal.pcbi.1005324.

Real-SPINE: Dor, O.; Zhou, Y. Real-SPINE: An Integrated System of Neural Networks for Real-Value Prediction of Protein Structural Properties. Proteins 2007, 68 (1), 76–81. https://doi.org/10.1002/prot.21408.

Real-SPINE2: Xue, B.; Dor, O.; Faraggi, E.; Zhou, Y. Real-Value Prediction of Backbone Torsion Angles. Proteins 2008, 72 (1), 427–433. https://doi.org/10.1002/prot.21940.

Real-SPINE3: Faraggi, E.; Xue, B.; Zhou, Y. Improving the Prediction Accuracy of Residue Solvent Accessibility and Real-Value Backbone Torsion Angles of Proteins by Guided-Learning through a Two-Layer Neural Network. Proteins 2009, 74 (4), 847–856. https://doi.org/10.1002/prot.22193.

RGN: AlQuraishi, M. End-to-End Differentiable Learning of Protein Structure. Cell Syst 2019, 8 (4), 292-301.e3. https://doi.org/10.1016/j.cels.2019.03.006.

RGN2: Chowdhury, R.; Bouatta, N.; Biswas, S.; Rochereau, C.; Church, G. M.; Sorger, P. K.; AlQuraishi, M. Single-Sequence Protein Structure Prediction Using Language Models from Deep Learning. bioRxiv August 4, 2021, p 2021.08.02.454840. https://doi.org/10.1101/2021.08.02.454840.

RNAcontact:Sun, S.; Wang, W.; Peng, Z.; Yang, J.; RNA inter-nucleotide 3D closeness prediction by deep residual neural networks. Bioinformatics, 2021, 37, 1093–1098.

Rosetta: Simons, K. T.; Kooperberg, C.; Huang, E.; Baker, D. Assembly of Protein Tertiary Structures from Fragments with Similar Local Sequences Using Simulated Annealing and Bayesian Scoring Functions11Edited by F. E. Cohen. Journal of Molecular Biology 1997, 268 (1), 209–225. https://doi.org/10.1006/jmbi.1997.0959.

Rosetta+NN: Meiler, J.; Baker, D. Coupled Prediction of Protein Secondary and Tertiary Structure. Proc Natl Acad Sci U S A 2003, 100 (21), 12105–12110. https://doi.org/10.1073/pnas.1831973100.

RoseTTAFold: Baek, M.; DiMaio, F.; Anishchenko, I.; Dauparas, J.; Ovchinnikov, S.; Lee, G. R.; Wang, J.; Cong, Q.; Kinch, L. N.; Schaeffer, R. D.; Millán, C.; Park, H.; Adams, C.; Glassman, C. R.; DeGiovanni, A.; Pereira, J. H.; Rodrigues, A. V.; van Dijk, A. A.; Ebrecht, A. C.; Opperman, D. J.; Sagmeister, T.; Buhlheller, C.; Pavkov-Keller, T.; Rathinaswamy, M. K.; Dalwadi, U.; Yip, C. K.; Burke, J. E.; Garcia, K. C.; Grishin, N. V.; Adams, P. D.; Read, R. J.; Baker, D. Accurate Prediction of Protein Structures and Interactions Using a 3-Track Neural Network. Science 2021, 373 (6557), 871–876. <https://doi.org/10.1126/science.abj8754>.

RoseTTAFold2: Baek, M.; Anishchenko, I.; Humphreys, I. R.; Cong, Qian, Baker, D., DiMaio, F., Efficient and accurate prediction of protein structure using RoseTTAFold2, bioRxiv, May 25, 2023, doi: https://doi.org/10.1101/2023.05.24.542179.

S4PRED: Moffat, L.; Jones, D. T. Increasing the Accuracy of Single Sequence Prediction Methods Using a Deep Semi-Supervised Learning Framework. Bioinformatics 2021, 37 (21), 3744–3751. <https://doi.org/10.1093/bioinformatics/btab491>.

SAM-T98: Karplus, K.; Barrett, C.; Hughey, R. Hidden Markov Models for Detecting Remote Protein Homologies. Bioinformatics 1998, 14 (10), 846–856.

SAM-T02: Karplus, K.; Karchin, R.; Draper, J.; Casper, J.; Mandel-Gutfreund, Y.; Diekhans, M.; Hughey, R. Combining Local-Structure, Fold-Recognition, and New Fold Methods for Protein Structure Prediction. Proteins 2003, 53 Suppl 6, 491–496.

SimFold: Chikenji, G.; Fujitsuka, Y.; Takada, S. A Reversible Fragment Assembly Method for de Novo Protein Structure Prediction. J. Chem. Phys. 2003, 119 (13), 6895–6903. <https://doi.org/10.1063/1.1597474>.

SPARKS: Zhou, H.; Zhou, Y. Single-Body Residue-Level Knowledge-Based Energy Score Combined with Sequence-Profile and Secondary Structure Information for Fold Recognition. Proteins 2004, 55 (4), 1005–1013. https://doi.org/10.1002/prot.20007.

SPARKS-X: Yang, Y.; Faraggi, E.; Zhao, H.; Zhou, Y. Improving Protein Fold Recognition and Template-Based Modeling by Employing Probabilistic-Based Matching between Predicted One-Dimensional Structural Properties of Query and Corresponding Native Properties of Templates. Bioinformatics 2011, 27 (15), 2076–2082.

SPIDER: Lyons, J.; Dehzangi, A.; Heffernan, R.; Sharma, A.; Paliwal, K.; Sattar, A.; Zhou, Y.; Yang, Y. Predicting Backbone Cα Angles and Dihedrals from Protein Sequences by Stacked Sparse Auto-Encoder Deep Neural Network. J Comput Chem 2014, 35 (28), 2040–2046. https://doi.org/10.1002/jcc.23718.

SPIDER 2: Heffernan, R.; Paliwal, K.; Lyons, J.; Dehzangi, A.; Sharma, A.; Wang, J.; Sattar, A.; Yang, Y.; Zhou, Y. Improving Prediction of Secondary Structure, Local Backbone Angles, and Solvent Accessible Surface Area of Proteins by Iterative Deep Learning. Sci Rep 2015, 5, 11476. https://doi.org/10.1038/srep11476.

SPIDER 3: Heffernan, R.; Yang, Y.; Paliwal, K.; Zhou, Y. Capturing Non-Local Interactions by Long Short-Term Memory Bidirectional Recurrent Neural Networks for Improving Prediction of Protein Secondary Structure, Backbone Angles, Contact Numbers and Solvent Accessibility. Bioinformatics 2017, 33 (18), 2842–2849. https://doi.org/10.1093/bioinformatics/btx218.

SPIDER2-Grid: Gao, J.; Yang, Y.; Zhou, Y. Grid-Based Prediction of Torsion Angle Probabilities of Protein Backbone and Its Application to Discrimination of Protein Intrinsic Disorder Regions and Selection of Model Structures. BMC Bioinformatics 2018, 19 (1), 29. <https://doi.org/10.1186/s12859-018-2031-7>.

SPIDER3-Single: Heffernan, R.; Paliwal, K.; Lyons, J.; Singh, J.; Yang, Y.; Zhou, Y. Single-Sequence-Based Prediction of Protein Secondary Structures and Solvent Accessibility by Deep Whole-Sequence Learning. J Comput Chem 2018, 39 (26), 2210–2216. https://doi.org/10.1002/jcc.25534.

SPINE XI: Faraggi, E.; Yang, Y.; Zhang, S.; Zhou, Y. Predicting Continuous Local Structure and the Effect of Its Substitution for Secondary Structure in Fragment-Free Protein Structure Prediction. Structure 2009, 17 (11), 1515–1527. https://doi.org/10.1016/j.str.2009.09.006.

SPOT-1D: Hanson, J.; Paliwal, K.; Litfin, T.; Yang, Y.; Zhou, Y. Improving Prediction of Protein Secondary Structure, Backbone Angles, Solvent Accessibility and Contact Numbers by Using Predicted Contact Maps and an Ensemble of Recurrent and Residual Convolutional Neural Networks. Bioinformatics 2019, 35 (14), 2403–2410. https://doi.org/10.1093/bioinformatics/bty1006.

SPOT-1D-LM: Singh, J.; Paliwal, K.; Litfin, T.; Singh, J.; Zhou, Y. Reaching Alignment-Profile-Based Accuracy in Predicting Protein Secondary and Tertiary Structural Properties without Alignment. Sci Rep 2022, 12 (1), 7607. https://doi.org/10.1038/s41598-022-11684-w.

SPOT-1D-Single: Singh, J.; Litfin, T.; Paliwal, K.; Singh, J.; Hanumanthappa, A. K.; Zhou, Y. SPOT-1D-Single: Improving the Single-Sequence-Based Prediction of Protein Secondary Structure, Backbone Angles, Solvent Accessibility and Half-Sphere Exposures Using a Large Training Set and Ensembled Deep Learning. Bioinformatics 2021, btab316. https://doi.org/10.1093/bioinformatics/btab316.

SPOT-Fold: Cai, Y.; Li, X.; Sun, Z.; Lu, Y.; Zhao, H.; Hanson, J.; Paliwal, K.; Litfin, T.; Zhou, Y.; Yang, Y. SPOT-Fold: Fragment-Free Protein Structure Prediction Guided by Predicted Backbone Structure and Contact Map. J Comput Chem 2020, 41 (8), 745–750.

SPOT-RNA: Singh, J.; Hanson, J.; Paliwal, K.; Zhou, Y.; RNA secondary structure prediction using an ensemble of two-dimensional deep neural networks and transfer learning, Nature Communications 2019, 10, 5407.

SPOT-RNA2: Singh, J.; Paliwal, K.; Zhang, T.; Singh, J.; Litfin, T.; Zhou, Y.; Improved RNA secondary structure and tertiary base-pairing prediction using evolutionary profile, mutational coupling and two-dimensional transfer learning, Bioinformatics, 2021, 37, 2589–2600.

SPOT-RNA-2D: Singh, J.; Paliwal, K.; Litfin, T.; Singh, J.; Zhou, Y.; Predicting RNA distance-based contact maps by integrated deep learning on physics-inferred secondary structure and evolutionary- derived mutational coupling.”, Bioinformatics, 2022, 38, 3900-3910.

Swiss-Model: Peitsch, M. C. ProMod and Swiss-Model: Internet-Based Tools for Automated Comparative Protein Modelling. Biochem Soc Trans 1996, 24 (1), 274–279. <https://doi.org/10.1002/jcc.26132>.

TASSER: Zhang, Y.; Skolnick, J. Automated Structure Prediction of Weakly Homologous Proteins on a Genomic Scale. Proc Natl Acad Sci U S A 2004, 101 (20), 7594–7599.

Uni-Fold: Li, Z.; Liu, X.; Chen, W.; Bi, H.; Ke, G.; Zhang, L. Uni-Fold: An Open-Source Platform for Developing Protein Folding Models beyond AlphaFold. August 06, 2022. doi: https://doi.org/10.1101/2022.08.04.502811
